# Research progress on the multi-omics and survival status of circulating tumor cells

**DOI:** 10.1007/s10238-024-01309-z

**Published:** 2024-03-01

**Authors:** Qingming Xie, Shilei Liu, Sai Zhang, Liqiu Liao, Zhi Xiao, Shouman Wang, Pengfei Zhang

**Affiliations:** 1 NHC Key Laboratory of Cancer Proteomics, Department of Oncology, Xiangya Hospital, Central South University, Changsha, 410008 Hunan People’s Republic of China; 2 National Clinical Research Center for Geriatric Disorders, Xiangya Hospital, Central South University, Changsha, 410008 Hunan People’s Republic of China; 3 Department of Breast Surgery, Hunan Clinical Meditech Research Center for Breast Cancer, Xiangya Hospital, Central South University, Changsha, 410008 Hunan People’s Republic of China

**Keywords:** Blood microenvironment, CTCs, CTC clusters, EMT, Multi-omics

## Abstract

In the dynamic process of metastasis, circulating tumor cells (CTCs) emanate from the primary solid tumor and subsequently acquire the capacity to disengage from the basement membrane, facilitating their infiltration into the vascular system via the interstitial tissue. Given the pivotal role of CTCs in the intricate hematogenous metastasis, they have emerged as an essential resource for a deeper comprehension of cancer metastasis while also serving as a cornerstone for the development of new indicators for early cancer screening and new therapeutic targets. In the epoch of precision medicine, as CTC enrichment and separation technologies continually advance and reach full fruition, the domain of CTC research has transcended the mere straightforward detection and quantification. The rapid advancement of CTC analysis platforms has presented a compelling opportunity for in-depth exploration of CTCs within the bloodstream. Here, we provide an overview of the current status and research significance of multi-omics studies on CTCs, including genomics, transcriptomics, proteomics, and metabolomics. These studies have contributed to uncovering the unique heterogeneity of CTCs and identifying potential metastatic targets as well as specific recognition sites. We also review the impact of various states of CTCs in the bloodstream on their metastatic potential, such as clustered CTCs, interactions with other blood components, and the phenotypic states of CTCs after undergoing epithelial-mesenchymal transition (EMT). Within this context, we also discuss the therapeutic implications and potential of CTCs.

## Introduction

Cancer metastasis remains a major contributor to cancer-related mortality, making it a central focus of cancer research. CTCs, as precursors of metastasis, offer a valuable perspective for studying the metastatic process[1]. In the context of cancer prognosis, a burgeoning body of evidence highlights the significance of CTC count as a potent predictor. Investigations into breast cancer, colorectal cancer, lung cancer, and esophageal cancer have yielded compelling findings. The assessment of CTCs emerges as a prospective tool, offering invaluable prognostic insights for cancer patients across various stages of the disease spectrum, whether it be in the early phases, advanced stages, or spanning the critical phases before and after therapeutic interventions[2–7]. Hence, valuable information regarding potential factors leading to unfavorable patient prognoses should be found within CTCs. In cancer research, the direct and pivotal approach to unveiling the molecular characteristics of CTCs is through sequencing. In a single-cell sequencing research focused on CTCs in non-small cell lung cancer, it was discovered that CTCs harbor genetic mutations from both the primary tumor and metastatic lesions, encompassing mutations specific to both the primary tumor and metastatic sites [8]. Therefore, considering CTCs as an alternative subject of study for solid tumors, without the necessity of obtaining primary tumor tissue, to obtain partial information regarding the tumor, presents a feasible approach. Furthermore, valuable and unique data that is distinct from the primary tumor can be gathered through the analysis of CTCs. On one hand, as it can be detected at the early stage in many cancers [9–12], CTCs have the potential to enhance the early screening of cancer through the analysis of CTCs. For instance, cancer can be initially diagnosed through the recognition of CTC-specific antigens [13]. On the other hand, it can also contribute to a more profound understanding of the mechanisms underlying metastasis by studying CTCs. In this context, we elucidate the present status of multi-omics investigations on CTCs conducted in recent years. It is important to emphasize that the biological phenotype of tumor cells is the result of a complex interplay between genetics and the environment, including morphological and phenotypic states, and the specific environment in which CTCs exist determines their unique biological phenotype [14, 15]. Therefore, this review will present the latest research findings concerning the survival status of CTCs in the bloodstream in recent years. This includes the EMT phenotype, as well as the interactions of CTCs with other constituents of the blood and the molecular characteristics of CTC-clusters.

## Multi-omics studies of CTCs

### Genomic research of CTCs

Genomic research has played a crucial role in identifying genes that are specific or of particular significance in various types of cancer. And it has led to significant advancements in uncovering genetic mutations associated with cancer initiation and progression, encompassing mechanisms of pathogenesis, immune evasion, metastasis, proliferation, and resistance to apoptosis [16]. The genome of cancer is markedly unstable, which may be a major driver of cancer development [17, 18]. Certain gene mutations have been clinically validated to predict cancer prognosis, establish associations with chemotherapy or immunotherapy responsiveness, and facilitate the development of tailored treatment plans for individual variations [19–26]. However, unveiling the "veil" of the cancer metastasis mechanism cannot be achieved solely through the exploration of the genome of solid cancer [27]. Profiling the genome of CTCs reveals unique implications distinct from those of the primary tumor. Distant metastasis from primary tumors involves a genetic mutation process, with most distant metastases acquiring driving mutations that are absent in primary tumors [28]. The process of hematogenous metastasis entails genetic changes in tumor cells. In the research of metastatic breast cancer, certain mutations with targeted potential, such as *BRCA2* p.Q1931X and *PTCH1* p.E1242X mutations, were present in CTCs and remain undetected in the corresponding tumor tissue [29]. Similarly, in pancreatic ductal adenocarcinoma, the KRAS mutations present in CTCs are inconsistent with those observed in the primary tumors, indicating genetic differences in their characteristics [30]. In expanded genomic profiling of CTCs in metastatic breast cancer patients, differences in the mutation statuses of *ESR1* and *ERBB2* between CTCs and their matched primary tumors were observed [31]. CTCs genome also holds potential significance for personalized therapy. On the one hand, the presence of genetic mutations in CTCs exhibits pronounced inter-patient heterogeneity. In genomic analysis of CTCs in colorectal cancer, there were notable disparities in the mutation statuses of *KRAS*, *BRAF*, and *PIK3CA*, as well as variations in the amplification status of epidermal growth factor receptor-1 (EGFR), among CTCs from different patients. Consequently, CTCs exhibit considerable heterogeneity from one patient to another, underscoring their potential as a foundation for personalized cancer treatment [32]. The genomic research findings on CTCs in non-small cell lung cancer similarly support this conclusion [33]. One the other hand, gene mutations in CTCs have a certain impact on treatment. In a study of metastatic breast cancer, disparities were identified in the CTC mutation status before and after treatment. Remarkably, a small quantity of CTCs carrying specific mutations could remain survival after treatment. These findings indicate that CTCs with certain gene mutations may develop resistance to drugs [34]. Gene mutations in CTCs can be used to study resistance mechanisms or to develop targeted therapeutics [35]. The utilization of targeted medications against drug-resistant mutations in cancer has demonstrated some effectiveness [36]. Consequently, genomic studies of circulating tumor cells (CTCs) offer substantial potential not only in understanding their heterogeneity but also in refining treatment strategies to tackle the challenging of cancer drug resistance.

### Transcriptomic research of CTCs

Transcriptomic research serves as a vital bridge connecting the genome to the proteome. The analysis of the transcriptome is indispensable for understanding the intricacies of the genome, unveiling the molecular composition of tumor cells, and addressing the progression of diseases. Together, the genome and transcriptome provide a comprehensive perspective on individual cancer patients, significantly influencing clinical decisions [37]. Notably, the gene expression of CTCs does not align perfectly with that of the primary tumor. In certain breast cancer patients, markers such as EGFR, epidermal growth factor receptor-2 (HER-2), and estrogen receptor (ER) may exhibit a negative status in the primary tumor, but CTCs can still manifest positive expression [38–40]. It has been proven to have certain prognostic significance in breast cancer and bladder cancer [41, 42]. In CTCs with specific phenotypes, there are specific pathway activation that can induce CTC proliferation [38]. The gene expression profiles discussed above carry significant implications for therapeutic strategies, such as targeted therapies. Within CTCs, there exist numerous promising targets awaiting exploration. The incorporation of this data is anticipated to enhance the precision and efficacy of cancer treatment [43]. Through transcriptome sequencing, a wealth of metastasis-related information can be obtained from CTCs, enriching genes associated with enhanced tumor cell proliferation and increased invasive capacity. These are essential components in exploring the mechanism of tumor metastasis [44–46]. Among them, there are numerous potential therapeutic targets, various pro-cancer pathways or more invasive CTC subpopulations [47–51]. Given the substantial heterogeneity observed among tumor cells, the need for single-cell transcriptome studies focusing on CTCs is evident. Single-cell transcriptomics has proven its worth in investigating CTC heterogeneity [52], making it a promising avenue for identifying precise diagnostic and prognostic markers, as well as actionable therapeutic targets for personalized cancer treatments [53]. And single-cell analysis can provide unique insights into the mechanisms of metastatic colonization by CTCs and precision medicine for cancers [54, 55]. Rapidly developing single-cell isolation technologies include the MagSweeper device [56], laser capture microdissection [57], NanoVelcro-LMD technology [58] and manual cell picking through micromanipulators [59]. Single-cell technology now allows comprehensive analysis of CTCs to reveal aspects of metastasis and resistance mechanisms in cancer therapy [52]. Moreover, existing sequencing technologies such as Hydro-Seq [60], Smart-seq [61], and Smart-seq2 [62] gave a strong basis to the feasibility of individual CTCs for RNA-seq analysis, allowing contamination-free high-throughput and sensitive analysis of the transcriptome of CTCs. Substantial progress has been achieved in revealing the heterogeneity of CTCs using single-cell transcriptomics. By comparing the single-cell RNA-sequencing results of CTCs from different vascular sites, including the hepatic vein, peripheral artery, peripheral vein and portal vein, in patients with hepatocellular carcinoma, Sun YF et al. found there was significant heterogeneity in CTCs between different vascular sites and different patients, involving differences in gene expression related to immunity, oxidative phosphorylation/metabolism, G protein-coupled receptor signaling, cell cycle, EMT, tumor cell-platelet microaggregates, and immune-suppressive chemokines [63, 64]. In the study of CTCs in breast cancer, CTCs in the blood from patients at different time stages (including active and dormant phases) as well as from mouse models show differential expression of cytokinesis and mitotic genes such as Ki67 [65]. Therefore, transcriptome sequencing for CTCs needs to be more systematic, taking into account time and space. Single-cell RNA sequencing (scRNA-seq) for CTCs is also an important source of information for metastatic targets and drug resistance mechanisms. The results of scRNA-seq on individual CTCs isolated from patients with pancreatic cancer, breast cancer, and prostate cancer have revealed that extracellular matrix (ECM) protein genes are highly expressed in human CTCs [66]. Considering the role of ECM lectins in cancer metastasis [67, 68], and the high expression of ECM gene family members in CTCs from several cancer types,  their upregulation may play a crucial role in the generation of CTCs from primary tumors. ScRNA-seq analysis of prostate cancer CTCs by David T. Miyamoto has shown that CTCs exhibit various alterations in androgen receptors (AR), including AR splice variants and point mutations, which are associated with clinical resistance to anti-androgen therapy [46]. In pancreatic ductal adenocarcinoma, scRNA-seq analysis has revealed that the expression of apoptosis inhibitor protein family member BIRC5 (survivin) is higher in CTCs compared to the primary tumor. This may be closely related to the survival mechanisms of CTCs in the bloodstream. Therefore, BIRC5 may be a significant target for reducing metastasis [69].

### Proteomic research of CTCs

At present, research on CTCs primarily focuses on genomics and transcriptomics. However, various modifications of proteins are necessary during the translation process, and transcriptomic data cannot fully reflect the qualitative and quantitative changes in protein expression during tumor development [70, 71]. Moreover, transcriptome and protein expression are not completely correlated [72–74]. Typically, single-cell proteomics analysis methods offer higher precision compared to current scRNA-seq methods. Proteomics technology enables high-throughput and rapid analysis of various proteins expressed in tumors, making it possible to identify numerous protein markers with diagnostic value [75, 76]. These proteins may provide new targets for tumor treatment and effective tumor biomarkers for early diagnosis. However, the limited number of CTCs in peripheral blood, the complexity of protein types within single cells, low protein content, and non-amplifiability pose challenges to CTC proteomics research. Mass spectrometry-based single-cell proteomics research on CTCs has not been reported yet. Nevertheless, some studies have identified some of the proteins present on CTCs by specific means, such as targeting assays. Single-cell resolution western blotting have identified glyceraldehyde-3-phosphate dehydrogenase, β-microtubulin, pankeratin, extracellular regulated protein kinases, epithelial cell adhesion molecule (EpCAM), ER, eIF6E and other proteins expressed in CTCs derived from breast cancer patients [77]. Reza KK et al. successfully detected the heterogeneity of melanoma CTCs and changes in biomarkers after treatment by a surface-enhanced Raman spectroscopy-based simple microfluidic device [78]. The studies described above were based on targeted detection of known proteins and could not detect the possible presence of unknown proteins in CTCs, which may have implications for metastatic drivers, tumor markers, and therapeutic targets. Single-cell proteomics based on mass spectrometry is rapidly advancing, allowing for the identification of biomarkers and the uncovering of heterogeneity within CTCs [79–83]. Therefore, proteomic studies of CTCs are very promising.

### Metabolomic research of CTCs

Metabolomics is the analytical research of small molecule metabolites, similar to other "histological" techniques, which can provide critical information about the status of cancers [84]. The metabolic status of cancers is available to differentiate cancer cell subtypes [85], identify cancer biomarkers and drivers of tumorigenesis and can also provide advice for targeted therapies [86, 87]. Tumor cells can adapt to the complex microenvironment and promote immune escape by altering metabolic patterns or reprogramming multiple metabolic pathways [88]. Thus, CTCs in the circulation should have a unique and suitable metabolic pattern. Due to the trace presence of CTCs in the blood, the identification of CTC metabolites poses a formidable challenge. Studies on the metabolomics of CTCs are currently limited to the validation of the presence of known metabolic pathways in CTCs, such as through gene expression assays or performing in vitro cultures of CTC lineage cells to gain insight into the metabolism of CTCs [89–91]. The in-depth exploration of CTCs metabolomics remains a significant gap in current research. The limited presence of CTCs in the bloodstream poses a formidable challenge for the non-targeted identification of their metabolites. On one front, it is focused on the intracellular metabolites of CTCs. Currently, single-cell metabolomics based on mass spectrometry is rapidly advancing. Takayuki Kawai integrated capillary electrophoresis with mass spectrometry and identified 40 metabolites from single HeLa cell [92]. Shuting Xu and colleagues developed a multidimensional organic mass cytometry platform based on chip-nanoelectrospray ionization mass cytometry system, capable of providing 100 metabolic parameters at single-cell resolution [93]. The use of magnetic bead separation combined with downstream laser desorption/ionization mass spectrometry has been developed and holds potential for identifying metabolic products in CTCs [94]. However, the metabolic products secreted by CTCs and the influence of the blood microenvironment on CTCs are factors that cannot be ignored in the study of CTC metabolism. This includes factors such as blood flow shear stress, oxygen levels, nutrients, and the impact of immune cells on CTCs. Simulating the microenvironment in which CTCs exist in the blood is essential. Currently, relevant research on simulating the blood environment has made some progress, including the simulation and real-time monitoring of hemodynamics, which involves shear stress, blood oxygen transport, and content [95–99], as well as red blood cell simulation [100]. The nutritional gradients in the blood are also a critical factor to consider. Moreover, in blood simulations, it may be necessary to extract and culture white blood cells from patients’ blood for blood simulation [101]. In summary, metabolomics is rapidly advancing, and despite facing numerous challenges in the field of CTCs metabolomics research, with continuous technological progress, the study of CTCs metabolism is gradually becoming feasible and holds tremendous potential in addressing the challenges of cancer metastasis.

## Clustered CTCs

### Study methods and prognostic significance of CTC clusters

CTC clusters are rare multicellular populations that can be detected in the early stages of cancers such as pancreatic cancer [10], breast cancer [11] and small cell lung cancer [12]. This implied that the transmission of CTC clusters is an early event in the cancer progression. Cohort studies across different cancer types have consistently shown that patients with CTC clusters tend to have significantly shorter survival compared to those without CTC clusters. Analyzing CTC clusters not only provides valuable prognostic information beyond individual CTC counts but also reveals a correlation between the size of CTC clusters and patient outcomes. Larger CTC clusters are associated with a higher risk of death [102–108]. The existence of CTC clusters is a significant factor that impacts the prognosis of cancer patients, making it a highly valuable area of research. The analysis of CTC clusters was also based on their enrichment and separation, and many CTC isolation techniques were incompatible with CTC cluster isolation. In recent years, techniques for CTC clusters have developed rapidly. The two-stage sequential microfluidic chip invented by Sam H. Au et al. could successively capture CTC clusters with less cell damage based on the larger size and asymmetry of CTC clusters [109]. The ScreenCell Cyto-Cl filter could separate individual CTCs and CTC clusters in a single blood sample [110], and the NanoVelcro chip based on EpCAM enrichment with densely packed silicon nanowires could capture CTC clusters with low EpCAM expression [111].

### Molecular properties of CTC clusters

Research by Nicola Aceto et al. in breast cancer revealed that CTC clusters originate from the primary tumor and are held together by plakoglobin proteins, which are found at bridging granules and adhesion junctions. [112, 113]. In colorectal cancer research, CTC clusters were also considered to be directly released from the solid tumor [114]. The formation of CTC clusters in hepatocellular carcinoma has shown correlation with the activation of the Wnt/β-catenin signaling pathway [115]. Xia Liu et al. observed by intravital multiphoton microscopic imaging in human MDA-MD-231 cell-derived tumor model mouse vasculature that individual CTCs could gather in clusters in the bloodstream and demonstrated that this process is inseparable from the role of CD44 [116]. Additionally, in breast cancer, the appearance of CTC clusters is closely related to intratumor hypoxia [117]. In several studies, the formation and metastatic potential of CTC clusters were also found to be regulated by acetyl heparinase and intercellular cell adhesion molecule-1 (ICAM); among them, ICAM was also found to have the ability to mediate CTC transendothelialization [118, 119]. Also, in breast cancer, disseminated tumor cell clusters are mainly composed of keratin 14-expressing cells[120]. In terms of genetics, genetic analysis of CTC clusters in breast cancer showed that CTC clusters were significantly associated with cadherin 1 (CDH1) [121] and *ESR1* Y537S and D538G mutations [122]. CDH1 is a gene located on chromosome 16 that encodes E-calmodulin, a member of the classical calmodulin family [123]. Taken together, formation of CTC clusters is regulated by various factors, including genetic mutations, the action of various adhesion factors, and the activation of signaling pathways. (Fig. 2). However, the current research on the molecular characteristics of CTC clusters is largely concentrated in breast cancer. The complexity of the formation mechanism of CTC clusters implies that current studies are relatively limited. The presence of CTC clusters has been confirmed in various cancers, highlighting the importance of focusing on the diversity and heterogeneity across different cancer types.

### CTC clusters grasp the advantage

CTC cluster size will not hinder their capacity to reach metastatic sites via capillaries. [124]. In breast cancer, compared to single CTCs, binding sites for stemness- and proliferation-associated transcription factors, including *OCT4*, *NANOG*, *SOX2*, and *SIN3A*, are specifically hypomethylated in the methylation region of CTC cluster DNA [125], which may confer enhanced metastatic and proliferative potential to CTC clusters. Furthermore, combination and collaboration among CTCs within the clusters could provide CTC clusters with an advantage through blood flow stresses, such as loss of nest apoptosis, shear force and immune attack, and colonization of distant organs [126]. In the mouse breast cancer model, there is an increase in the intercellular adhesion and epithelial gene expression of CTC clusters. This leads to a decrease in the expression of the activation ligands for NK cells, conferring low sensitivity of CTC clusters to NK-mediated suppression [127].

### Potential therapeutic value in CTC clusters

Clinical follow-up studies on colorectal cancer and hepatocellular carcinoma suggest that chemotherapy cannot completely eliminate CTC clusters [128, 129], which leads to potential risks of recurrence and metastasis. The persistence of CTC clusters significantly increases the probability of metastasis. Therefore, treatment targeting CTC clusters is of crucial importance in reducing the occurrence of metastasis and improving prognosis. This can be approached from two perspectives: firstly, disassembling CTC clusters. As mentioned above, the formation of CTC clusters is regulated by various factors in some cancers, and targeting the facilitators of CTC cluster formation may help reduce their occurrence or facilitate their disassembly. One study showed that urokinase-type fibrinogen activators had an inhibitory effect on CTC clusters and had the potential to improve survival in a mouse model of lung metastasis [130]. Na/K-ATPase inhibitors, such as ouabain and digitoxin (DT), could dissociate CTC clusters by increasing the intracellular calcium concentration, resulting in the inhibition of cell–cell junctions, and have the ability to inhibit the spontaneous shedding of CTC clusters from cancerous lesions and reduce the ability of cancer metastatic seeding [125]. What is more, the anti-EGFR monoclonal antibody (clone LA1) blocking EGFR has been shown to effectively inhibit in vitro the CD44-mediated aggregation of triple-negative breast cancer cells, reducing metastasis [131]. The use of celecoxib can reduce the formation of CTC clusters by inhibiting COX-2 and downregulating E-cadherin protein expression within xenograft tumors [132]. On the other hand, during tumor cell aggregation, it induced a hypoxic environment, including mitochondrial autophagy mediated by hypoxia-inducible factor 1-alpha (Hif1α) and restriction of reactive oxygen species (ROS). This ultimately reduces dependence on glycolysis for ATP production. Disrupting these metabolic adaptations may have the potential to decrease the survival and metastatic capability of tumor cells [133]. This is limited to cell line, and further expansion is needed to delve into research involving CTC clusters sourced directly from patients. However, treatment targeting CTC clusters also requires the development of efficient and convenient clinical detection methods [134]. Additionally, translating research into clinical applications necessitates a deeper understanding of the molecular characteristics of CTC clusters in different cancers. Clusters of CTCs in different types of cancer may harbor specific cancer-associated molecular characteristics. For instance, this could potentially offer more comprehensive guidance for the treatment of CTC clusters [135]. Treating solely targeting the CTC clusters may not achieve a significant enough effect, while combining with other treatment methods such as chemotherapy may lead to more pronounced outcomes [136]. The therapeutic potential and clinical applicability of identified targets also require further assessment.

## CTCs and EMT

**Fig. 1:**
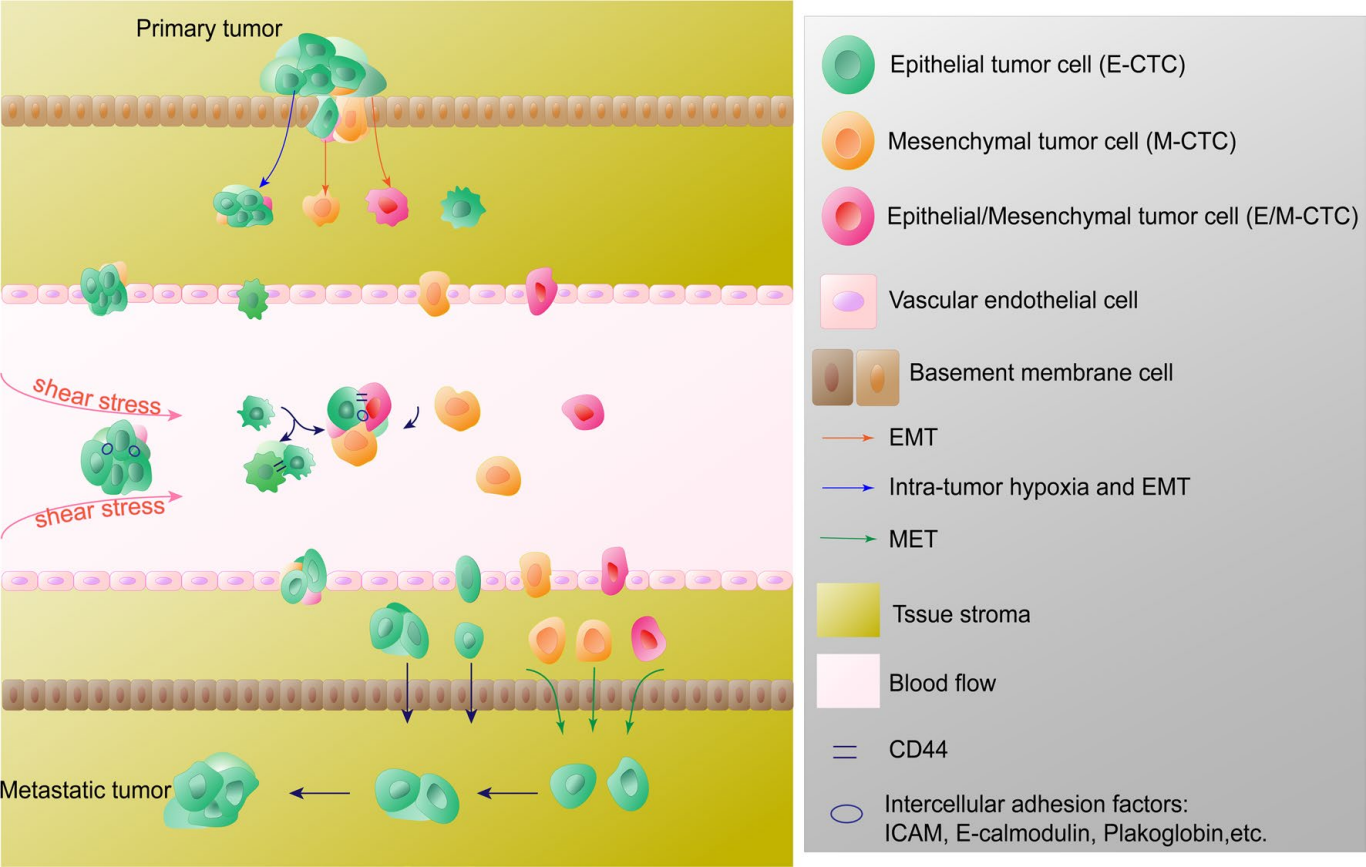
Partial tumor cells of the primary tumor, after undergoing or not undergoing complete or incomplete EMT, acquire a mesenchymal phenotype, either completely or incompletely, and invade into the circulation singly or in clusters with high expression of various epithelial adhesion factors by shedding from the primary tumor. They may also aggregate into clusters in the vasculature mediated by CD44. Clustered CTCs had an advantage in resistance to shear and had a higher metastatic and invasive potential. CTCs also undergo MET during metastatic colonization to acquire the property of adhering to the epithelium

In cancers, EMT plays a role in tumorigenesis, invasion and metastasis [137, 138]. Although the necessity of EMT in cancer metastasis remains controversial [139–141], its role in metastasis cannot be ignored. EMT studies of CTCs would more visually reflect the impact of EMT on metastasis and its therapeutic significance for CTCs. EMT is a complex process where tumor cells gradually shed their epithelial characteristics, leading to reduced intercellular adhesion. They then partially or completely acquire migratory and invasive properties, often resulting in the presentation of epithelial (E), mesenchymal (M), or epithelial/mesenchymal (E/M) hybrid phenotypes (Fig. 1). This transformation is accompanied by a decrease in the expression of epithelial markers such as EpCAM and E-calmodulin, while there is an increase in mesenchymal markers like vimentin, twist, and others. In the clinic, phenotypic detection of CTCs has shown potential in monitoring treatment resistance and prognosis prediction in cancer patients [142]. It has been found that human breast cancer cells and lung cancer cells with E/M hybrid phenotypes have stronger proliferative and invasive abilities [143, 144]. The value of E/M-CTCs in predicting adverse outcomes in patients and the occurrence of metastases has been confirmed in cohort studies of colorectal carcinoma [145], non-small cell lung carcinoma [146], and nasopharyngeal carcinoma [147]. There was also some clinical significance of M-CTCs for monitoring treatment response. In hepatocellular carcinoma [148], breast cancers [149] and gastric cancers [150], the proportion of M-CTCs decreased in patients whose treatment was effective, while the proportion of M-CTCs increased in the blood of patients whose disease progressed during treatment, and in pancreatic cancer, the percentage of M-CTCs was also more common in patients with advanced disease [151]. EMT may be involved in the immune evasion process of CTCs. In gastric cancer, CTCs that underwent EMT (E/M-CTC, M-CTC) were accompanied by downregulation of *ULBP1*, which facilitated its immune evasion from natural killer cells (NK cells). [152, 153]. The EMT status of CTCs was influenced by various factors. One study found that tumor-associated macrophages induced the EMT program by regulating the JAK2/STAT3/miR-506-3p/FoxQ1 axis, which increased the proportion of M-CTCs [154]. Platelet-derived TGFβ can activate the TGFβ/Smad and NF-κB pathways in cancer cells, resulting in their transition to an invasive M phenotype and enhanced metastasis in vivo [155]. Then, CTCs undergo MET when they reach the colonization site, and tumor cells with a mesenchymal phenotype regain their epithelial morphology, gain the ability to colonize, and reestablish intercellular connections to gradually form metastases [156].

## CTCs and blood microenvironment

**Fig. 2:**
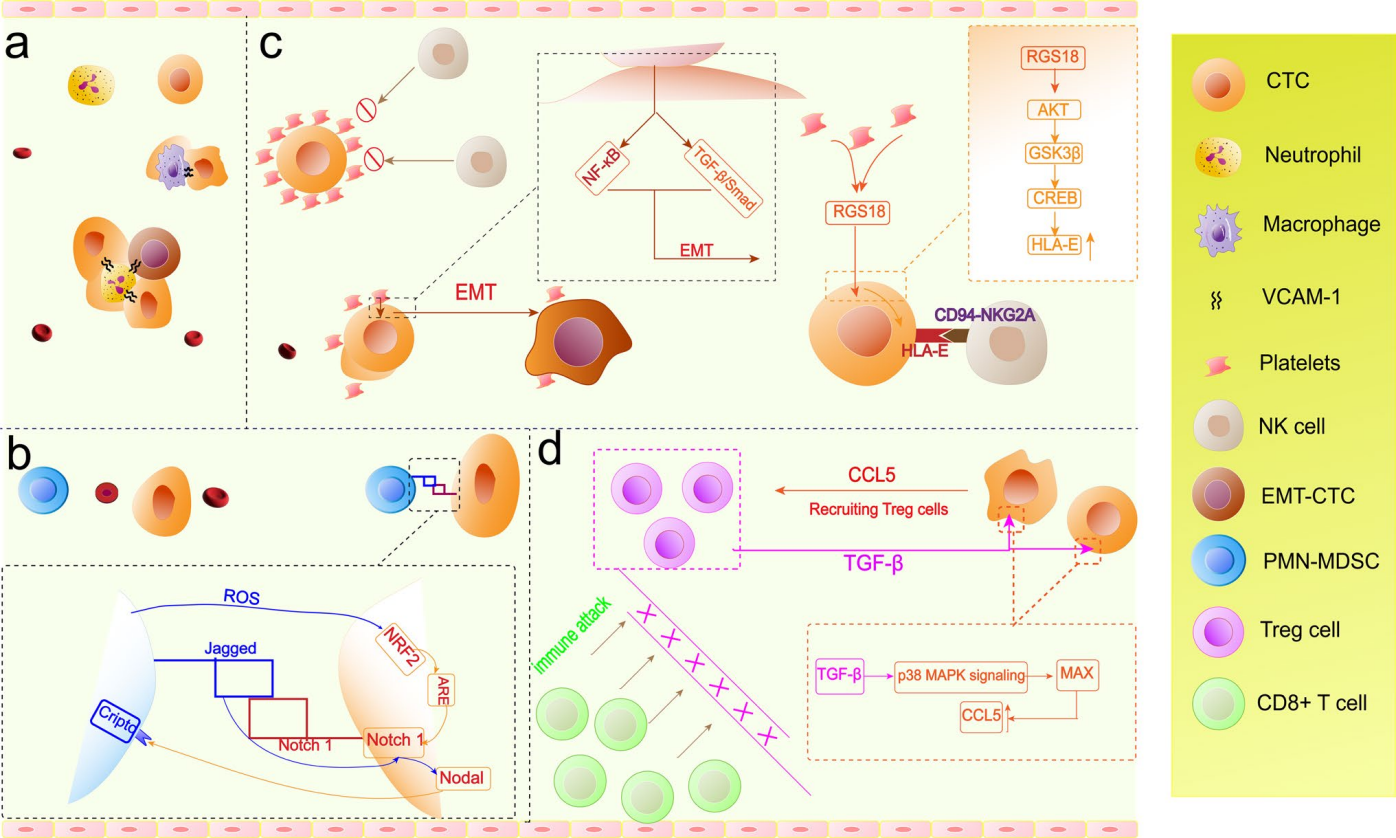
There were complex interactions between CTCs and other blood components, such as neutrophils, macrophages, and platelets, in the circulation, including aggregation into clusters and secretion of related cytokines, which contributed to a more potent invasive immune evasion ability for CTCs

Most CTCs are killed during their transmission in the bloodstream due to multiple stresses imposed by the blood microenvironment, including immune attack, shear, hypoxia, and attachment loss, but a few CTCs also respond to these negative effects by interacting with the blood microenvironment in which they are located, and this interaction also promotes the metastatic potential of CTCs (Fig. 2). On the one hand, CTCs can form heterotypic CTC clusters by binding to white blood cells (WBCs) or platelets in the blood, as found by Qiong Luo et al. in a study of hepatocellular carcinoma. Different CTC subtypes, including E-CTCs, M-CTCs and E/M-CTCs, can form CTC-associated WBC (CTC-WBC) clusters. CTC-WBC clusters are independent predictors of disease-free survival (DFS) and OS, and their presence indicates poor prognosis in patients with hepatocellular carcinoma [157]. Similarly, CTC-WBC clusters are also considered prognostic factors for advanced NSCLC [158] and renal cell carcinoma [159]. In renal cell carcinoma [160] and gastric cancer [161] after radical surgery and hormone receptor (HR)-positive/HER2-negative metastatic breast cancer receiving first-line chemotherapy with docetaxel plus capecitabine, CTC-WBC predicted a poor prognosis [162].

In breast cancer, CTCs from CTCs associated with neutrophil or macrophage clusters with a predominance of neutrophils mediated by vascular cell adhesion molecule-1 (VCAM1), CTCs in clusters exhibited upregulation of positive regulators of the cell cycle and DNA replication program and increased mutation frequency compared to individual CTCs, suggesting that direct interaction of CTCs with neutrophils increased the likelihood of metastatic colonization CTCs [163]. Marc L. Sprouse et al. isolated polymorphonuclear myeloid-derived suppressor cells (PMN-MDSCs) as well as CTCs and cocultured PMN-MDSCs with CTCs and human brain metastatic breast cancer cells (Luc-MDA-MB-231BR) or melanoma (Luc-70 W-SM3) cells and found that CTCs and tumor cells could form heterotypic clusters with PMN-MDSCs and that PMN-MDSC-cocultured tumor cells exhibited relatively high expression of Ki67. In in vitro experiments, CTCs formed clusters with PMN-MDSC and induced their pro-tumor differentiation via the paracrine nodal signal Nodal, which increased the production of reactive oxygen species (ROS) by PMN-MDSCs, and ROS upregulated Notch2 receptor expression in CTCs via the ROS-NRF1-ARE axis to promote tumor cell growth [101]. Although the researchers did not capture the CTC-PMN- MDSC clusters directly from the blood of cancer patients, the high levels of serum Nodal and ROS and high expression of *Nodal* in CTCs and the protumorigenic effect of MDSCs [164] implied that the interaction between these two types of cells is of great significance for cancer metastasis. Platelets and tumor metastasis were found to have a deep connection in earlier studies, with many complex interactions [165]. In mouse models, it was found that platelets can form a physical barrier through fibrin microthrombi for tumor cells [166], aggregate around tumor cells [167], or confer downregulated MHC class I [168] to tumor cells to evade the immune surveillance and killing effect from NK cells. Platelets could also establish direct contact with tumor cells and then lead to their transformation into an aggressive mesenchymal-like phenotype and enhance metastasis by activating the TGFβ/Smad and NF-κB pathways in cancer cells [155]. In CT26 mouse models of colorectal cancer, CTCs associated with platelets would promote their capture by neutrophil extracellular traps (NETs) and subsequent distant metastasis [169]. Similarly, CTCs can also be captured by NETs through interactions between NETs and CTCs mediated by β1-integrin released by neutrophils [170]. In pancreatic ductal adenocarcinoma, CTCs increase the expression of regulator of G-protein signaling 18 (RGS18) under the influence of platelets, and RGS18 can upregulate human leukocyte antigen-E (HLA-E) expression via the AKT-GSK3β-CREB signaling pathway. The upregulated HLA-E could interact with CD94-NKG2A on NK cells, which can protect CTCs from NK-mediated immune surveillance [171]. On the other hand, CTCs can interact with cells in the blood through relevant cytokines. It is worth mentioning that there is also an indirect interaction between blood components and CTCs. In hepatocellular carcinoma CTCs, there is overexpression of *CCL5*, an immunosuppressive chemokine that recruits regulatory T cells (Treg cells), which could suppress abnormal immune responses against their own antigens and antitumor immune responses [172] and then promote the immune escape of tumors. CTCs recruit Treg cells via CCL5 to establish a metastasis-friendly microenvironment during hematogenous metastasis(Fig. 2). Treg-derived TGF-β1 induces CCL5 production via p38-MAX signaling [64]. In terms of therapies, it was noted that multifunctional S-nitrosocaptopril acts on CTCs and platelets to interrupt platelet-CTC interactions and adhesion to the endothelium, thereby inhibiting lung metastasis in CT26 colon carcinoma mouse models [173].

## CTCs and targeted therapy

To date, CTCs remain key to the prevention and treatment of metastasis, whether they are the previously mentioned mutation sites of CTCs, metastasis-driven targets from transcriptome sequencing, attachment site CTC clusters, and pathways of CTCs interacting with the blood microenvironment that contain promising therapeutic targets. Targeted therapy against specific sites of CTCs is feasible. In metastatic breast cancer, after targeted therapeutic treatment implemented against HER-2, the number of CTCs in the patient's blood decreased [174]. This indicated that CTCs were responsive to targeted therapy. Weiwei Mu et al. developed a multipoint costriking nanodevice called GV-Lipo/sorafenib (SF)/DT. Anti-glypican-3 (GPC3) mAb and anti-VCAM1 mAb were modified on the surface of GV-Lipos. Among them, anti-GPC3 mAb recognized GPC3, a tumorigenic fetal proteoglycan with high specific expression in HCC, to capture GPC3-positive HCC CTCs, while anti-VCAM1 mAb captured CTC-neutrophil clusters, and then DT dissociated the clusters. Finally, the tumor cells would be killed by SF [175]. Similar to this, for instance, a dual-targeted nanoparticle therapy with paclitaxel as a model drug combined the tumor neoplastic targeting ligand K237 peptide with Ep23 aptamer into a single drug-loaded nanoplatform aiming at simultaneously destroying the primary tumor and neutralizing CTCs for synergistic anti-breast cancer treatment, which has been validated in a mouse model of breast cancer [176]. A nanosized neutrophil mimetic drug delivery system has also been developed to capture CTCs based on the adhesion of neutrophils to CTCs. In brief, treatment against CTCs has multidirectional options, deserving more focus and clinical trials.

## Discussion

In the era of precision medicine, it is imperative to broaden the scope of cancer research, with a particular emphasis on the pivotal role of CTCs in the metastasis cascade. Multi-omics research on CTCs has made certain progress in unraveling the mechanisms of cancer drug resistance and the driving factors behind the metastasis of various cancers. Nevertheless, there remains a wealth of untapped information awaiting exploration. Regrettably, the study of CTCs faces substantial technical challenges, particularly in the fields of proteomics and metabolomics. Proteomic and metabolic investigations are fundamental to comprehending cancer, and they necessitate further in-depth investigation. Furthermore, CTCs exhibit diverse survival states while in circulation, including the formation of homotypic and heterotypic clusters, as well as phenotypic changes following the EMT. These factors profoundly affect survival capabilities of CTCs, influencing aspects such as immune evasion, invasive potential, and proliferation. They also hold promise for identifying targets for metastatic treatment. The ultimate objective of CTC research is to enhance clinical practice. In one aspect, CTCs possess the potential to serve as early screening indicators for various cancers and as markers for minimal metastatic disease. CTCs have been found to emerge in the early stages of cancer, providing an opportunity for cancer screening in conditions where diagnosis may be challenging through other methods by detecting CTCs in the blood. Due to the short lifespan of CTCs in circulation, those detected years later are likely to originate from small micro-metastatic lesions. The determination of specific biomarkers for different types of cancer and distinct subtypes within the same cancer relies on multi-omics research. Additionally, CTCs have been associated with poor prognoses in numerous cancer types. Therefore, its feasibility as a prognostic marker is evident, underscoring the necessity for therapeutic strategies aimed at CTCs. Tackling this challenge demands a profound comprehension of the intricate heterogeneity of CTCs and their various survival states. However, the genuine clinical translation of CTC research still faces a series of challenges. Firstly, the detection of CTCs is a pivotal issue. Despite the plethora of methods available for CTC detection, the variations in sensitivity and specificity make standardization difficult. Currently, the only FDA-approved clinical device is the CellSearch system [177]. Nevertheless, this system is limited to capturing epithelial markers, resulting in the inability to capture CTCs that have undergone EMT, leading to the loss of CTCs with certain phenotypic information. The limitation also diminishes the prognostic value of CTC counting based on the CellSearch system. Therefore, the primary challenge in applying CTCs to clinical practice is the development of more comprehensive and standardized detection methods. As mentioned earlier, CTCs exhibit significant heterogeneity among different types of cancers and even among individuals with the same cancer type. Consequently, when applying CTCs to treatment, it is imperative to consider inter-individual differences, including variations in drug resistance due to genetic mutations, phenotypic differences, and the presence of CTC clusters. This underscores the necessity for efficient, convenient, and practical detection of CTCs and CTC clusters, whether in terms of cell counting, phenotyping, genetic mutation analysis, or target identification, to support clinical services in future. Furthermore, in the realm of CTC-targeted therapy, the development of treatment targets must take into account the therapeutic efficacy of specific targets in research. This involves a thorough understanding of the biological characteristics of different CTC subpopulations to formulate personalized treatment strategies, maximizing the potential for therapeutic effectiveness. It is also necessary to thoroughly consider the therapeutic effects against characteristic targets in studies. In summary, CTC omics studies have shed light on issues related to drug resistance and metastasis, but there is still much more to explore to advance cancer research and improve clinical outcomes.

## Data Availability

Not applicable.

## Abbreviations

AR: Androgen receptor
CTC-WBCs: CTC-associated WBCs
CTCs: Circulating tumor cells
DT: Digitoxin
E: Epithelial
E/M: Epithelial/mesenchymal
ECM: Extracellular matrix
EGFR: Epidermal growth factor receptor
EMT: Epithelial-mesenchymal transition
ER: Estrogen receptor
GPC3: Glypican-3
HER-2: Epidermal growth factor receptor-2
HLA-E: Human leukocyte antigen
ICAM: Intercellular cell adhesion molecule-1
M: Mesenchymal
MDSC: Myeloid-derived suppressor cells
NK cells: Natural killer cells
NETs: Neutrophil extracellular traps
PMN-MDSC: Polymorphonuclear-myeloid-derived suppressor cells
RNA-seq: RNA sequencing
ROS: Reactive oxygen species
scRNA-seq: Single-cell RNA sequencing
SF: Sorafenib
WBC: White blood cells
WNT: Wingless/integrated

## References

[1] Massagué J, Obenauf AC. Metastatic colonization. Nature. 2016;529:298–306. 26791720 10.1038/nature17038 PMC5029466

[2] Cristofanilli M, Budd GT, Ellis MJ, Stopeck A, Matera J, Miller MC, et al. Circulating tumor cells, disease progression, and survival in metastatic breast cancer. N Engl J Med. 2004;351:781–91. 15317891 10.1056/NEJMoa040766

[3] Lindsay CR, Faugeroux V, Michiels S, Pailler E, Facchinetti F, Ou D, et al. A prospective examination of circulating tumor cell profiles in non-small-cell lung cancer molecular subgroups. Ann Oncol. 2017;28:1523–31. 28633480 10.1093/annonc/mdx156

[4] Lucci A, Hall CS, Lodhi AK, Bhattacharyya A, Anderson AE, Xiao L, et al. Circulating tumour cells in non-metastatic breast cancer: a prospective study. Lancet Oncol. 2012;13:688–95. 22677156 10.1016/S1470-2045(12)70209-7

[5] Bian J, Yan K, Liu N, Xu X. Correlations between circulating tumor cell phenotyping and 18F-fluorodeoxyglucose positron emission tomography uptake in non-small cell lung cancer. J Cancer Res Clin Oncol. 2020;146:2621–30. 32661602 10.1007/s00432-020-03244-4 PMC11804374

[6] Flatmark K, Borgen E, Nesland JM, Rasmussen H, Johannessen H-O, Bukholm I, et al. Disseminated tumour cells as a prognostic biomarker in colorectal cancer. Br J Cancer. 2011;104:1434–9. 21448171 10.1038/bjc.2011.97 PMC3101945

[7] Bidard F-C, Michiels S, Riethdorf S, Mueller V, Esserman LJ, Lucci A, et al. Circulating tumor cells in breast cancer patients treated by neoadjuvant chemotherapy: a meta-analysis. J Natl Cancer Inst. 2018;110:560–7. 29659933 10.1093/jnci/djy018

[8] Su Z, Wang Z, Ni X, Duan J, Gao Y, Zhuo M, et al. Inferring the evolution and progression of small-cell lung cancer by single-cell sequencing of circulating tumor cells. Clin Cancer Res. 2019;25:5049–60. 31113842 10.1158/1078-0432.CCR-18-3571

[9] Zhang Z, Shiratsuchi H, Lin J, Chen G, Reddy RM, Azizi E, et al. Expansion of CTCs from early stage lung cancer patients using a microfluidic co-culture model. Oncotarget. 2014;5:12383–97. 25474037 10.18632/oncotarget.2592 PMC4323004

[10] Padillo-Ruiz J, Suarez G, Pereira S, Calero-Castro FJ, Tinoco J, Marin L, et al. Circulating tumor cells enumeration from the portal vein for risk stratification in early pancreatic cancer patients. Cancers (Basel). 2021;13:6153. 34944773 10.3390/cancers13246153 PMC8699156

[11] Reduzzi C, Di Cosimo S, Gerratana L, Motta R, Martinetti A, Vingiani A, et al. Circulating tumor cell clusters are frequently detected in women with early-stage breast cancer. Cancers (Basel). 2021;13:2356. 34068368 10.3390/cancers13102356 PMC8153325

[12] Murlidhar V, Reddy RM, Fouladdel S, Zhao L, Ishikawa MK, Grabauskiene S, et al. Poor prognosis indicated by venous circulating tumor cell clusters in early-stage lung cancers. Cancer Res. 2017;77:5194–206. 28716896 10.1158/0008-5472.CAN-16-2072 PMC5600850

[13] Lu S-H, Tsai W-S, Chang Y-H, Chou T-Y, Pang S-T, Lin P-H, et al. Identifying cancer origin using circulating tumor cells. Cancer Biol Ther. 2016;17:430–8. 26828696 10.1080/15384047.2016.1141839 PMC4910938

[14] Park JH, Pyun WY, Park HW. Cancer metabolism: phenotype. Signal Therap Targets Cells. 2020;9:2308. 10.3390/cells9102308 PMC7602974 33081387

[15] Annabi B, Rojas-Sutterlin S, Laflamme C, Lachambre M-P, Rolland Y, Sartelet H, et al. Tumor Environment Dictates Medulloblastoma Cancer Stem Cell Expression and Invasive Phenotype. Mol Cancer Res. 2008;6:907–16. 18567795 10.1158/1541-7786.MCR-07-2184

[16] Lawrence MS, Stojanov P, Mermel CH, Garraway LA, Golub TR, Meyerson M, et al. Discovery and saturation analysis of cancer genes across 21 tumor types. Nature. 2014;505:495–501. 24390350 10.1038/nature12912 PMC4048962

[17] Lee J-K, Choi Y-L, Kwon M, Park PJ. Mechanisms and consequences of cancer genome instability: lessons from genome sequencing studies. Annu Rev Pathol. 2016;11:283–312. 26907526 10.1146/annurev-pathol-012615-044446

[18] Lengauer C, Kinzler KW, Vogelstein B. Genetic instabilities in human cancers. Nature. 1998;396:643–9. 9872311 10.1038/25292

[19] Taron M, Ichinose Y, Rosell R, Mok T, Massuti B, Zamora L, et al. Activating mutations in the tyrosine kinase domain of the epidermal growth factor receptor are associated with improved survival in gefitinib-treated chemorefractory lung adenocarcinomas. Clin Cancer Res. 2005;11:5878–85. 16115929 10.1158/1078-0432.CCR-04-2618

[20] Chou T-Y, Chiu C-H, Li L-H, Hsiao C-Y, Tzen C-Y, Chang K-T, et al. Mutation in the tyrosine kinase domain of epidermal growth factor receptor is a predictive and prognostic factor for gefitinib treatment in patients with non-small cell lung cancer. Clin Cancer Res. 2005;11:3750–7. 15897572 10.1158/1078-0432.CCR-04-1981

[21] Concin N, Hofstetter G, Berger A, Gehmacher A, Reimer D, Watrowski R, et al. Clinical relevance of dominant-negative p73 isoforms for responsiveness to chemotherapy and survival in ovarian cancer: evidence for a crucial p53–p73 cross-talk in vivo. Clin Cancer Res. 2005;11:8372–83. 16322298 10.1158/1078-0432.CCR-05-0899

[22] Wang J, Yao Z, Jonsson P, Allen AN, Qin ACR, Uddin S, et al. A secondary mutation in BRAF confers resistance to RAF inhibition in a BRAF V600E-mutant brain tumor. Cancer Discov. 2018;8:1130–41. 29880583 10.1158/2159-8290.CD-17-1263 PMC6125191

[23] Wang Z, Zhang Q, Qi C, Bai Y, Zhao F, Chen H, et al. Combination of AKT1 and CDH1 mutations predicts primary resistance to immunotherapy in dMMR/MSI-H gastrointestinal cancer. J Immunother Cancer. 2022;10: e004703. 35705314 10.1136/jitc-2022-004703 PMC9204428

[24] Hu J, Cao J, Topatana W, Juengpanich S, Li S, Zhang B, et al. Targeting mutant p53 for cancer therapy: direct and indirect strategies. J Hematol Oncol. 2021;14:157. 34583722 10.1186/s13045-021-01169-0 PMC8480024

[25] Sicklick JK, Kato S, Okamura R, Schwaederle M, Hahn ME, Williams CB, et al. Molecular profiling of cancer patients enables personalized combination therapy: the I-PREDICT study. Nat Med. 2019;25:744–50. 31011206 10.1038/s41591-019-0407-5 PMC6553618

[26] Morganti S, Tarantino P, Ferraro E, D’Amico P, Viale G, Trapani D, et al. Complexity of genome sequencing and reporting: Next generation sequencing (NGS) technologies and implementation of precision medicine in real life. Crit Rev Oncol Hematol. 2019;133:171–82. 30661654 10.1016/j.critrevonc.2018.11.008

[27] Vogelstein B, Papadopoulos N, Velculescu VE, Zhou S, Diaz LA, Kinzler KW. Cancer genome landscapes. Science. 2013;339:1546–58. 23539594 10.1126/science.1235122 PMC3749880

[28] Yates LR, Knappskog S, Wedge D, Farmery JHR, Gonzalez S, Martincorena I, et al. Genomic evolution of breast cancer metastasis and relapse. Cancer Cell. 2017;32:169-184.e7. 28810143 10.1016/j.ccell.2017.07.005 PMC5559645

[29] Paoletti C, Cani AK, Larios JM, Hovelson DH, Aung K, Darga EP, et al. Comprehensive mutation and copy number profiling in archived circulating breast cancer tumor cells documents heterogeneous resistance mechanisms. Cancer Res. 2018;78:1110–22. 29233927 10.1158/0008-5472.CAN-17-2686 PMC5815882

[30] Kulemann B, Rösch S, Seifert S, Timme S, Bronsert P, Seifert G, et al. Pancreatic cancer: circulating tumor cells and primary tumors show heterogeneous KRAS mutations. Sci Rep. 2017;7:4510. 28674438 10.1038/s41598-017-04601-z PMC5495768

[31] Magbanua MJM, Rugo HS, Wolf DM, Hauranieh L, Roy R, Pendyala P, et al. Expanded genomic profiling of circulating tumor cells in metastatic breast cancer patients to assess biomarker status and biology over time (CALGB 40502 and CALGB 40503, Alliance). Clin Cancer Res. 2018;24:1486–99. 29311117 10.1158/1078-0432.CCR-17-2312 PMC5856614

[32] Gasch C, Bauernhofer T, Pichler M, Langer-Freitag S, Reeh M, Seifert AM, et al. Heterogeneity of epidermal growth factor receptor status and mutations of KRAS/PIK3CA in circulating tumor cells of patients with colorectal cancer. Clin Chem. 2013;59:252–60. 23136247 10.1373/clinchem.2012.188557

[33] Owen S, Lo T-W, Fouladdel S, Zeinali M, Keller E, Azizi E, et al. Simultaneous single cell gene expression and EGFR mutation analysis of circulating tumor cells reveals distinct phenotypes in NSCLC. Adv Biosyst. 2020;4: e2000110. 32700450 10.1002/adbi.202000110 PMC7883301

[34] De Luca F, Rotunno G, Salvianti F, Galardi F, Pestrin M, Gabellini S, et al. Mutational analysis of single circulating tumor cells by next generation sequencing in metastatic breast cancer. Oncotarget. 2016;7:26107–19. 27034166 10.18632/oncotarget.8431 PMC5041968

[35] Carter L, Rothwell DG, Mesquita B, Smowton C, Leong HS, Fernandez-Gutierrez F, et al. Molecular analysis of circulating tumor cells identifies distinct copy-number profiles in patients with chemosensitive and chemorefractory small-cell lung cancer. Nat Med. 2017;23:114–9. 27869802 10.1038/nm.4239

[36] Sequist LV, Soria J-C, Goldman JW, Wakelee HA, Gadgeel SM, Varga A, et al. Rociletinib in EGFR-mutated non-small-cell lung cancer. N Engl J Med. 2015;372:1700–9. 25923550 10.1056/NEJMoa1413654

[37] Roychowdhury S, Chinnaiyan AM. Translating cancer genomes and transcriptomes for precision oncology. CA Cancer J Clin. 2016;66:75–88. 26528881 10.3322/caac.21329 PMC4713245

[38] Kallergi G, Agelaki S, Kalykaki A, Stournaras C, Mavroudis D, Georgoulias V. Phosphorylated EGFR and PI3K/Akt signaling kinases are expressed in circulating tumor cells of breast cancer patients. Breast Cancer Res. 2008;10:R80. 18822183 10.1186/bcr2149 PMC2614515

[39] Beije N, Onstenk W, Kraan J, Sieuwerts AM, Hamberg P, Dirix LY, et al. Prognostic impact of HER2 and ER status of circulating tumor cells in metastatic breast cancer patients with a HER2-negative primary tumor. Neoplasia. 2016;18:647–53. 27764697 10.1016/j.neo.2016.08.007 PMC5071539

[40] Boral D, Vishnoi M, Liu HN, Yin W, Sprouse ML, Scamardo A, et al. Molecular characterization of breast cancer CTCs associated with brain metastasis. Nat Commun. 2017;8:196. 28775303 10.1038/s41467-017-00196-1 PMC5543046

[41] Wallwiener M, Hartkopf AD, Riethdorf S, Nees J, Sprick MR, Schönfisch B, et al. The impact of HER2 phenotype of circulating tumor cells in metastatic breast cancer: a retrospective study in 107 patients. BMC Cancer. 2015;15:403. 25972110 10.1186/s12885-015-1423-6 PMC4435916

[42] Rink M, Chun FK, Dahlem R, Soave A, Minner S, Hansen J, et al. Prognostic role and HER2 expression of circulating tumor cells in peripheral blood of patients prior to radical cystectomy: a prospective study. Eur Urol. 2012;61:810–7. 22277196 10.1016/j.eururo.2012.01.017

[43] Rupp B, Ball H, Wuchu F, Nagrath D, Nagrath S. Circulating tumor cells in precision medicine: challenges and opportunities. Trends Pharmacol Sci. 2022;43:378–91. 35272862 10.1016/j.tips.2022.02.005

[44] Franses JW, Philipp J, Missios P, Bhan I, Liu A, Yashaswini C, et al. Pancreatic circulating tumor cell profiling identifies LIN28B as a metastasis driver and drug target. Nat Commun. 2020;11:3303. 32620742 10.1038/s41467-020-17150-3 PMC7335061

[45] Yu M, Ting DT, Stott SL, Wittner BS, Ozsolak F, Paul S, et al. RNA sequencing of pancreatic circulating tumour cells implicates WNT signaling in metastasis. Nature. 2012;487:510–3. 22763454 10.1038/nature11217 PMC3408856

[46] Miyamoto DT, Zheng Y, Wittner BS, Lee RJ, Zhu H, Broderick KT, et al. RNA-seq of single prostate CTCs implicates noncanonical Wnt signaling in antiandrogen resistance. Science. 2015;349:1351–6. 26383955 10.1126/science.aab0917 PMC4872391

[47] Lin Z, Radaeva M, Cherkasov A, Dong X. Lin28 regulates cancer cell stemness for tumour progression. Cancers (Basel). 2022;14:4640. 36230562 10.3390/cancers14194640 PMC9564245

[48] Lovnicki J, Gan Y, Feng T, Li Y, Xie N, Ho C-H, et al. LIN28B promotes the development of neuroendocrine prostate cancer. J Clin Invest. 2020;130:5338–48. 32634132 10.1172/JCI135373 PMC7524485

[49] Molenaar JJ, Domingo-Fernández R, Ebus ME, Lindner S, Koster J, Drabek K, et al. LIN28B induces neuroblastoma and enhances MYCN levels via let-7 suppression. Nat Genet. 2012;44:1199–206. 23042116 10.1038/ng.2436

[50] Manier S, Powers JT, Sacco A, Glavey SV, Huynh D, Reagan MR, et al. The LIN28B/let-7 axis is a novel therapeutic pathway in multiple myeloma. Leukemia. 2017;31:853–60. 27773931 10.1038/leu.2016.296 PMC5382134

[51] Ebright RY, Lee S, Wittner BS, Niederhoffer KL, Nicholson BT, Bardia A, et al. Deregulation of ribosomal protein expression and translation promotes breast cancer metastasis. Science. 2020;367:1468–73. 32029688 10.1126/science.aay0939 PMC7307008

[52] Keller L, Pantel K. Unravelling tumour heterogeneity by single-cell profiling of circulating tumour cells. Nat Rev Cancer. 2019;19:553–67. 31455893 10.1038/s41568-019-0180-2

[53] Zhang S, Wei JS, Khan J. Chapter 4 - The Significance of Transcriptome Sequencing in Personalized Cancer Medicine. In: Dellaire G, Berman JasonN, Arceci RJ, editors. Cancer Genomics [Internet]. Boston: Academic Press; 2014 [cited 2023 Oct 13]. p. 49–64. Available from: https://www.sciencedirect.com/science/article/pii/B9780123969675000049

[54] Gohil SH, Iorgulescu JB, Braun DA, Keskin DB, Livak KJ. Applying high-dimensional single-cell technologies to the analysis of cancer immunotherapy. Nat Rev Clin Oncol. 2021;18:244–56. 33277626 10.1038/s41571-020-00449-x PMC8415132

[55] Tieng FYF, Baharudin R, Abu N, Mohd Yunos R-I, Lee L-H, Ab Mutalib N-S. Single Cell Transcriptome in Colorectal Cancer—Current Updates on Its Application in Metastasis, Chemoresistance and the Roles of Circulating Tumor Cells. Frontiers in Pharmacology [Internet]. 2020 [cited 2023 Mar 27];11. Available from: https://www.frontiersin.org/articles/10.3389/fphar.2020.00135 10.3389/fphar.2020.00135 PMC7056698 32174835

[56] Talasaz AH, Powell AA, Huber DE, Berbee JG, Roh K-H, Yu W, et al. Isolating highly enriched populations of circulating epithelial cells and other rare cells from blood using a magnetic sweeper device. Proc Natl Acad Sci U S A. 2009;106:3970–5. 19234122 10.1073/pnas.0813188106 PMC2645911

[57] Jan YJ, Chen J-F, Zhu Y, Lu Y-T, Chen SH, Chung H, et al. NanoVelcro rare-cell assays for detection and characterization of circulating tumor cells. Adv Drug Deliv Rev. 2018;125:78–93. 29551650 10.1016/j.addr.2018.03.006 PMC5993593

[58] Lin M, Chen J-F, Lu Y-T, Zhang Y, Song J, Hou S, et al. Nanostructure embedded microchips for detection, isolation, and characterization of circulating tumor cells. Acc Chem Res. 2014;47:2941–50. 25111636 10.1021/ar5001617 PMC4204926

[59] Lohr JG, Adalsteinsson VA, Cibulskis K, Choudhury AD, Rosenberg M, Cruz-Gordillo P, et al. Whole-exome sequencing of circulating tumor cells provides a window into metastatic prostate cancer. Nat Biotechnol. 2014;32:479–84. 24752078 10.1038/nbt.2892 PMC4034575

[60] Cheng Y-H, Chen Y-C, Lin E, Brien R, Jung S, Chen Y-T, et al. Hydro-Seq enables contamination-free high-throughput single-cell RNA-sequencing for circulating tumor cells. Nat Commun. 2019;10:2163. 31092822 10.1038/s41467-019-10122-2 PMC6520360

[61] Ramsköld D, Luo S, Wang Y-C, Li R, Deng Q, Faridani OR, et al. Full-Length mRNA-Seq from single cell levels of RNA and individual circulating tumor cells. Nat Biotechnol. 2012;30:777–82. 22820318 10.1038/nbt.2282 PMC3467340

[62] Picelli S, Faridani OR, Björklund AK, Winberg G, Sagasser S, Sandberg R. Full-length RNA-seq from single cells using Smart-seq2. Nat Protoc. 2014;9:171–81. 24385147 10.1038/nprot.2014.006

[63] Sun Y-F, Guo W, Xu Y, Shi Y-H, Gong Z-J, Ji Y, et al. Circulating tumor cells from different vascular sites exhibit spatial heterogeneity in epithelial and mesenchymal composition and distinct clinical significance in hepatocellular carcinoma. Clin Cancer Res. 2018;24:547–59. 29070526 10.1158/1078-0432.CCR-17-1063

[64] Sun Y-F, Wu L, Liu S-P, Jiang M-M, Hu B, Zhou K-Q, et al. Dissecting spatial heterogeneity and the immune-evasion mechanism of CTCs by single-cell RNA-seq in hepatocellular carcinoma. Nat Commun. 2021;12:4091. 34215748 10.1038/s41467-021-24386-0 PMC8253833

[65] Diamantopoulou Z, Castro-Giner F, Schwab FD, Foerster C, Saini M, Budinjas S, et al. The metastatic spread of breast cancer accelerates during sleep. Nature. 2022;607:156–62. 35732738 10.1038/s41586-022-04875-y

[66] Ting DT, Wittner BS, Ligorio M, Jordan NV, Shah AM, Miyamoto DT, et al. Single-cell RNA sequencing identifies extracellular matrix gene expression by pancreatic circulating tumor cells. Cell Rep. 2014;8:1905–18. 25242334 10.1016/j.celrep.2014.08.029 PMC4230325

[67] Talmadge JE, Fidler IJ. AACR centennial series: the biology of cancer metastasis: historical perspective. Cancer Res. 2010;70:5649–69. 20610625 10.1158/0008-5472.CAN-10-1040 PMC4037932

[68] Su Z, Yang Z, Xu Y, Chen Y, Yu Q. Apoptosis, autophagy, necroptosis, and cancer metastasis. Molecular Cancer [Internet]. 2015 [cited 2023 Apr 2];14. Available from: https://www.ncbi.nlm.nih.gov/pmc/articles/PMC4343053/10.1186/s12943-015-0321-5 PMC4343053 25743109

[69] Li F, Aljahdali I, Ling X. Cancer therapeutics using survivin BIRC5 as a target: what can we do after over two decades of study? J Exp Clin Cancer Res. 2019;38:368. 31439015 10.1186/s13046-019-1362-1 PMC6704566

[70] Wang D, Eraslan B, Wieland T, Hallström B, Hopf T, Zolg DP, et al. A deep proteome and transcriptome abundance atlas of 29 healthy human tissues. Mol Syst Biol. 2019;15: e8503. 30777892 10.15252/msb.20188503 PMC6379049

[71] Albayrak C, Jordi CA, Zechner C, Lin J, Bichsel CA, Khammash M, et al. Digital quantification of proteins and mRNA in single mammalian cells. Mol Cell. 2016;61:914–24. 26990994 10.1016/j.molcel.2016.02.030

[72] Buccitelli C, Selbach M. mRNAs, proteins and the emerging principles of gene expression control. Nat Rev Genet. 2020;21:630–44. 32709985 10.1038/s41576-020-0258-4

[73] Washburn MP, Koller A, Oshiro G, Ulaszek RR, Plouffe D, Deciu C, et al. Protein pathway and complex clustering of correlated mRNA and protein expression analyses in Saccharomyces cerevisiae. Proc Natl Acad Sci U S A. 2003;100:3107–12. 12626741 10.1073/pnas.0634629100 PMC152254

[74] Zhang B, Wang J, Wang X, Zhu J, Liu Q, Shi Z, et al. Proteogenomic characterization of human colon and rectal cancer. Nature. 2014;513:382–7. 25043054 10.1038/nature13438 PMC4249766

[75] Gonçalves E, Poulos RC, Cai Z, Barthorpe S, Manda SS, Lucas N, et al. Pan-cancer proteomic map of 949 human cell lines. Cancer Cell. 2022;40:835-849.e8. 35839778 10.1016/j.ccell.2022.06.010 PMC9387775

[76] Huang Z, Ma L, Huang C, Li Q, Nice EC. Proteomic profiling of human plasma for cancer biomarker discovery. Proteomics. 2017;17. 10.1002/pmic.201600240 27550791

[77] Sinkala E, Sollier-Christen E, Renier C, Rosàs-Canyelles E, Che J, Heirich K, et al. Profiling protein expression in circulating tumour cells using microfluidic western blotting. Nat Commun. 2017;8:14622. 28332571 10.1038/ncomms14622 PMC5376644

[78] Reza KK, Dey S, Wuethrich A, Wang J, Behren A, Antaw F, Wang Y, Sina AA, Trau M. In situ single cell proteomics reveals circulating tumor cell heterogeneity during treatment. ACS Nano. 2021;15(7):11231–43. 34225455 10.1021/acsnano.0c10008

[79] Bennett HM, Stephenson W, Rose CM, Darmanis S. Single-cell proteomics enabled by next-generation sequencing or mass spectrometry. Nat Methods. 2023;20:363–74. 36864196 10.1038/s41592-023-01791-5

[80] Tajik M, Baharfar M, Donald WA. Single-cell mass spectrometry. Trends Biotechnol. 2022;40:1374–92. 35562238 10.1016/j.tibtech.2022.04.004

[81] Zhu Y, Clair G, Chrisler WB, Shen Y, Zhao R, Shukla AK, et al. Proteomic analysis of single mammalian cells enabled by microfluidic nanodroplet sample preparation and ultrasensitive nanoLC-MS. Angew Chem Int Ed Engl. 2018;57:12370–4. 29797682 10.1002/anie.201802843 PMC6261339

[82] Woo J, Williams SM, Markillie LM, Feng S, Tsai C-F, Aguilera-Vazquez V, et al. High-throughput and high-efficiency sample preparation for single-cell proteomics using a nested nanowell chip. Nat Commun. 2021;12:6246. 34716329 10.1038/s41467-021-26514-2 PMC8556371

[83] Petrosius V, Aragon-Fernandez P, Üresin N, Kovacs G, Phlairaharn T, Furtwängler B, et al. Exploration of cell state heterogeneity using single-cell proteomics through sensitivity-tailored data-independent acquisition. Nat Commun. 2023;14:5910. 37737208 10.1038/s41467-023-41602-1 PMC10517177

[84] Schmidt DR, Patel R, Kirsch DG, Lewis CA, Vander Heiden MG, Locasale JW. metabolomics in cancer research and emerging applications in clinical oncology. CA Cancer J Clin. 2021;71:333–58. 33982817 10.3322/caac.21670 PMC8298088

[85] Zhang W, Xu F, Yao J, Mao C, Zhu M, Qian M, et al. Single-cell metabolic fingerprints discover a cluster of circulating tumor cells with distinct metastatic potential. Nat Commun. 2023;14:2485. 37120634 10.1038/s41467-023-38009-3 PMC10148826

[86] Mm R, J I, M G, G S. Identification of bioactive metabolites using activity metabolomics. Nature reviews Molecular cell biology [Internet]. 2019 [cited 2023 Mar 31]; 20. Available from: https://pubmed.ncbi.nlm.nih.gov/30814649/10.1038/s41580-019-0108-4 PMC6613555 30814649

[87] Luengo A, Gui DY, Vander Heiden MG. Targeting metabolism for cancer therapy. Cell Chem Biol. 2017;24:1161–80. 28938091 10.1016/j.chembiol.2017.08.028 PMC5744685

[88] Wang XY, Zhang J, Li Y, He JM. Research progress on the regulation of tumor metabolism, tumor immunotherapy and new analytical methods. Acta Pharmaceutica Sinica. 2020:2080-91.

[89] Hong X, Roh W, Sullivan RJ, Wong KHK, Wittner BS, Guo H, et al. The lipogenic regulator SREBP2 induces transferrin in circulating melanoma cells and suppresses ferroptosis. Cancer Discov. 2021;11:678–95. 33203734 10.1158/2159-8290.CD-19-1500 PMC7933049

[90] Zhu Z, Achreja A, Meurs N, Animasahun O, Owen S, Mittal A, et al. Tumour-reprogrammed stromal BCAT1 fuels branched-chain ketoacid dependency in stromal-rich PDAC tumours. Nat Metab. 2020;2:775–92. 32694827 10.1038/s42255-020-0226-5 PMC7438275

[91] Hong X, Roh W, Sullivan RJ, Wong KHK, Wittner BS, Guo H, et al. The lipogenic regulator SREBF2 induces transferrin in circulating melanoma cells and suppresses ferroptosis. Cancer Discov. 2021;11:678–95. 33203734 10.1158/2159-8290.CD-19-1500 PMC7933049

[92] Kawai T, Ota N, Okada K, Imasato A, Owa Y, Morita M, et al. Ultrasensitive single cell metabolomics by capillary electrophoresis-mass spectrometry with a thin-walled tapered emitter and large-volume dual sample preconcentration. Anal Chem. 2019;91:10564–72. 31357863 10.1021/acs.analchem.9b01578

[93] Xu S, Liu M, Bai Y, Liu H. Multi-dimensional organic mass cytometry: simultaneous analysis of proteins and metabolites on single cells. Angew Chem Int Ed Engl. 2021;60:1806–12. 33085796 10.1002/anie.202009682

[94] Wu J, Wei X, Gan J, Huang L, Shen T, Lou J, et al. Multifunctional magnetic particles for combined circulating tumor cells isolation and cellular metabolism detection. Adv Funct Mater. 2016;26:4016–25. 27524958 10.1002/adfm.201504184 PMC4978350

[95] Wu T, Shen J, Li Z, Zou T, Xin W, Xing F, et al. Graphene-based ultrasensitive optical microfluidic sensor for the real-time and label-free monitoring of simulated arterial blood flow. Opt Express. 2020;28:16594–604. 32549478 10.1364/OE.392993

[96] Deng W, Tsubota K. Numerical simulation of the vascular structure dependence of blood flow in the kidney. Med Eng Phys. 2022;104: 103809. 35641074 10.1016/j.medengphy.2022.103809

[97] Causin P, Guidoboni G, Malgaroli F, Sacco R, Harris A. Blood flow mechanics and oxygen transport and delivery in the retinal microcirculation: multiscale mathematical modeling and numerical simulation. Biomech Model Mechanobiol. 2016;15:525–42. 26232093 10.1007/s10237-015-0708-7

[98] Celaya-Alcala JT, Lee GV, Smith AF, Li B, Sakadžić S, Boas DA, et al. Simulation of oxygen transport and estimation of tissue perfusion in extensive microvascular networks: Application to cerebral cortex. J Cereb Blood Flow Metab. 2021;41:656–69. 32501155 10.1177/0271678X20927100 PMC7922761

[99] Yu Z, Tan J, Wang S. Enhanced discrete phase model for multiphase flow simulation of blood flow with high shear stress. Sci Prog. 2021;104:368504211008064. 33788651 10.1177/00368504211008064 PMC10358624

[100] Ye T, Phan-Thien N, Lim CT. Particle-based simulations of red blood cells—A review. J Biomech. 2016;49:2255–66. 26706718 10.1016/j.jbiomech.2015.11.050

[101] Sprouse ML, Welte T, Boral D, Liu HN, Yin W, Vishnoi M, et al. PMN-MDSCs Enhance CTC Metastatic Properties through Reciprocal Interactions via ROS/Notch/Nodal Signaling. Int J Mol Sci. 2019;20:1916. 31003475 10.3390/ijms20081916 PMC6514876

[102] Costa C, Muinelo-Romay L, Cebey-López V, Pereira-Veiga T, Martínez-Pena I, Abreu M, et al. Analysis of a Real-World Cohort of Metastatic Breast Cancer Patients Shows Circulating Tumor Cell Clusters (CTC-clusters) as Predictors of Patient Outcomes. Cancers (Basel). 2020;12:1111. 32365530 10.3390/cancers12051111 PMC7281711

[103] Manjunath Y, Upparahalli SV, Suvilesh KN, Avella DM, Kimchi ET, Staveley-O’Carroll KF, et al. Circulating tumor cell clusters are a potential biomarker for detection of non-small cell lung cancer. Lung Cancer. 2019;134:147–50. 10.1016/j.lungcan.2019.06.016 31319973

[104] Wang C, Zhang Z, Chong W, Luo R, Myers RE, Gu J, et al. Improved Prognostic Stratification Using Circulating Tumor Cell Clusters in Patients with Metastatic Castration-Resistant Prostate Cancer. Cancers (Basel). 2021;13:268. 33450815 10.3390/cancers13020268 PMC7828213

[105] Larsson A-M, Jansson S, Bendahl P-O, Levin Tykjaer Jörgensen C, Loman N, Graffman C, et al. Longitudinal enumeration and cluster evaluation of circulating tumor cells improve prognostication for patients with newly diagnosed metastatic breast cancer in a prospective observational trial. Breast Cancer Res. 2018;20:48. 10.1186/s13058-018-0976-0 PMC5994056 29884204

[106] Murlidhar V, Reddy RM, Fouladdel S, Zhao L, Ishikawa MK, Grabauskiene S, et al. Poor Prognosis Indicated by Venous Circulating Tumor Cell Clusters in Early Stage Lung Cancers. Cancer Res. 2017;77:5194–206. 28716896 10.1158/0008-5472.CAN-16-2072 PMC5600850

[107] Wang C, Mu Z, Chervoneva I, Austin L, Ye Z, Rossi G, et al. Longitudinally collected CTCs and CTC-clusters and clinical outcomes of metastatic breast cancer. Breast Cancer Res Treat. 2017;161:83–94. 27771841 10.1007/s10549-016-4026-2

[108] Yu J-J, Shu C, Yang H-Y, Huang Z, Li Y-N, Tao R, et al. The Presence of Circulating Tumor Cell Cluster Characterizes an Aggressive Hepatocellular Carcinoma Subtype. Front Oncol. 2021;11: 734564. 34722281 10.3389/fonc.2021.734564 PMC8554092

[109] Au SH, Edd J, Stoddard AE, Wong KHK, Fachin F, Maheswaran S, et al. Microfluidic Isolation of Circulating Tumor Cell Clusters by Size and Asymmetry. Sci Rep. 2017;7:2433. 28550299 10.1038/s41598-017-01150-3 PMC5446400

[110] Francescangeli F, Magri V, De Angelis ML, De Renzi G, Gandini O, Zeuner A, et al. Sequential Isolation and Characterization of Single CTCs and Large CTC Clusters in Metastatic Colorectal Cancer Patients. Cancers (Basel). 2021;13:6362. 34944983 10.3390/cancers13246362 PMC8699456

[111] Sun N, Yang Y, Miao H, Redublo P, Liu H, Liu W, et al. Discovery and characterization of circulating tumor cell clusters in neuroendocrine tumor patients using nanosubstrate-embedded microchips. Biosens Bioelectron. 2022;199: 113854. 34896918 10.1016/j.bios.2021.113854 PMC8900541

[112] Aceto N, Bardia A, Miyamoto DT, Donaldson MC, Wittner BS, Spencer JA, et al. Circulating tumor cell clusters are oligoclonal precursors of breast cancer metastasis. Cell. 2014;158:1110–22. 25171411 10.1016/j.cell.2014.07.013 PMC4149753

[113] Cowin P, Kapprell HP, Franke WW, Tamkun J, Hynes RO. Plakoglobin: a protein common to different kinds of intercellular adhering junctions. Cell. 1986;46:1063–73. 3530498 10.1016/0092-8674(86)90706-3

[114] Cima I, Kong SL, Sengupta D, Tan IB, Phyo WM, Lee D, et al. Tumor-derived circulating endothelial cell clusters in colorectal cancer. Science Translational Medicine. 2016;8:345ra89–345ra89. 10.1126/scitranslmed.aad7369 27358499

[115] Yu J-J, Shu C, Yang H-Y, Huang Z, Li Y-N, Tao R, et al. The Presence of Circulating Tumor Cell Cluster Characterizes an Aggressive Hepatocellular Carcinoma Subtype. Frontiers in Oncology [Internet]. 2021 [cited 2024 Jan 15];11. Available from: https://www.ncbi.nlm.nih.gov/pmc/articles/PMC8554092/10.3389/fonc.2021.734564 PMC8554092 34722281

[116] Liu X, Taftaf R, Kawaguchi M, Chang Y-F, Chen W, Entenberg D, et al. Homophilic CD44 interactions mediate tumor cell aggregation and polyclonal metastasis in patient-derived breast cancer models. Cancer Discov. 2019;9:96–113. 30361447 10.1158/2159-8290.CD-18-0065 PMC6328322

[117] Donato C, Kunz L, Castro-Giner F, Paasinen-Sohns A, Strittmatter K, Szczerba BM, et al. Hypoxia Triggers the Intravasation of Clustered Circulating Tumor Cells. Cell Rep. 2020;32: 108105. 32905777 10.1016/j.celrep.2020.108105 PMC7487783

[118] Wei R, Sun D, Yang H, Yan J, Zhang X, Zheng X, et al. CTC clusters induced by heparanase enhance breast cancer metastasis. Acta Pharmacol Sin. 2018;39:1326–37. 29417941 10.1038/aps.2017.189 PMC6289387

[119] Taftaf R, Liu X, Singh S, Jia Y, Dashzeveg NK, Hoffmann AD, et al. ICAM1 initiates CTC cluster formation and trans-endothelial migration in lung metastasis of breast cancer. Nature Communications [Internet]. 2021 [cited 2023 Mar 21];12. Available from: https://www.ncbi.nlm.nih.gov/pmc/articles/PMC8358026/10.1038/s41467-021-25189-z PMC8358026 34381029

[120] Cheung KJ, Padmanaban V, Silvestri V, Schipper K, Cohen JD, Fairchild AN, et al. Polyclonal breast cancer metastases arise from collective dissemination of keratin 14-expressing tumor cell clusters. Proc Natl Acad Sci U S A. 2016;113:E854–63. 26831077 10.1073/pnas.1508541113 PMC4763783

[121] Davis AA, Zhang Q, Gerratana L, Shah AN, Zhan Y, Qiang W, et al. Association of a novel circulating tumor DNA next-generating sequencing platform with circulating tumor cells (CTCs) and CTC clusters in metastatic breast cancer. Breast Cancer Res. 2019;21:137. 31801599 10.1186/s13058-019-1229-6 PMC6894208

[122] Li Z, Wu Y, Yates ME, Tasdemir N, Bahreini A, Chen J, et al. Hotspot ESR1 mutations are multimodal and contextual modulators of breast cancer metastasis. Cancer Res. 2022;82:1321–39. 35078818 10.1158/0008-5472.CAN-21-2576 PMC8983597

[123] Huntsman DG, Caldas C. Assignment1 of the E-cadherin gene (CDH1) to chromosome 16q22.1 by radiation hybrid mapping. Cytogenet Cell Genet. 1998;83:82–3. 10.1159/000015134 9925936

[124] Sh A, Bd S, Jc M, Q T, Yl C, S J, et al. Clusters of circulating tumor cells traverse capillary-sized vessels. Proceedings of the National Academy of Sciences of the United States of America [Internet]. 2016 [cited 2023 Mar 22];113. Available from: https://pubmed.ncbi.nlm.nih.gov/27091969/10.1073/pnas.1524448113 PMC4983862 27091969

[125] Gkountela S, Castro-Giner F, Szczerba BM, Vetter M, Landin J, Scherrer R, et al. Circulating Tumor Cell Clustering Shapes DNA Methylation to Enable Metastasis Seeding. Cell. 2019;176:98-112.e14. 30633912 10.1016/j.cell.2018.11.046 PMC6363966

[126] Giuliano M, Shaikh A, Lo HC, Arpino G, De Placido S, Zhang XH, et al. Perspective on Circulating Tumor Cell Clusters: Why It Takes a Village to Metastasize. Can Res. 2018;78:845–52. 10.1158/0008-5472.CAN-17-2748 29437766

[127] Lo HC, Xu Z, Kim IS, Pingel B, Aguirre S, Kodali S, et al. Resistance to natural killer cell immunosurveillance confers a selective advantage to polyclonal metastasis. Nat Cancer. 2020;1:709–22. 35122036 10.1038/s43018-020-0068-9

[128] Molnar B, Ladanyi A, Tanko L, Sréter L, Tulassay Z. Circulating tumor cell clusters in the peripheral blood of colorectal cancer patients. Clin Cancer Res. 2001;7:4080–5. 11751505

[129] Amado V, González-Rubio S, Zamora J, Alejandre R, Espejo-Cruz ML, Linares C, et al. Clearance of circulating tumor cells in patients with hepatocellular carcinoma undergoing surgical resection or liver transplantation. Cancers (Basel). 2021;13:2476. 34069569 10.3390/cancers13102476 PMC8160727

[130] Choi JW, Kim JK, Yang YJ, Kim P, Yoon K-H, Yun SH. Urokinase exerts antimetastatic effects by dissociating clusters of circulating tumor cells. Can Res. 2015;75:4474–82. 10.1158/0008-5472.CAN-15-0684 26527605

[131] Liu X, Adorno-Cruz V, Chang Y-F, Jia Y, Kawaguchi M, Dashzeveg NK, et al. EGFR inhibition blocks cancer stem cell clustering and lung metastasis of triple negative breast cancer. Theranostics. 2021;11:6632–43. 33995681 10.7150/thno.57706 PMC8120216

[132] Balamurugan K, Poria DK, Sehareen SW, Krishnamurthy S, Tang W, McKennett L, et al. Stabilization of E-cadherin adhesions by COX-2/GSK3*β* signaling is a targetable pathway in metastatic breast cancer. JCI Insight. 2023;8:156057. 10.1172/jci.insight.156057 PMC10070121 36757813

[133] Labuschagne CF, Cheung EC, Blagih J, Domart M-C, Vousden KH. Cell clustering promotes a metabolic switch that supports metastatic colonization. Cell Metab. 2019;30:720-734.e5. 31447323 10.1016/j.cmet.2019.07.014 PMC6863392

[134] Boya M, Ozkaya-Ahmadov T, Swain BE, Chu C-H, Asmare N, Civelekoglu O, et al. High throughput, label-free isolation of circulating tumor cell clusters in meshed microwells. Nat Commun. 2022;13:3385. 35697674 10.1038/s41467-022-31009-9 PMC9192591

[135] Lu L, Hu W, Liu B, Yang T. Insights into circulating tumor cell clusters: a barometer for treatment effects and prognosis for prostate cancer patients. Cancers (Basel). 2022;14:3985. 36010983 10.3390/cancers14163985 PMC9406494

[136] Dashzeveg NK, Jia Y, Zhang Y, Gerratana L, Patel P, Shajahan A, et al. Dynamic glycoprotein hyposialylation promotes chemotherapy evasion and metastatic seeding of quiescent circulating tumor cell clusters in breast cancer. Cancer Discov. 2023;13:2050–71. 37272843 10.1158/2159-8290.CD-22-0644 PMC10481132

[137] Puisieux A, Brabletz T, Caramel J. Oncogenic roles of EMT-inducing transcription factors. Nat Cell Biol. 2014;16:488–94. 24875735 10.1038/ncb2976

[138] Brabletz T. To differentiate or not—routes towards metastasis. Nat Rev Cancer. 2012;12:425–36. 22576165 10.1038/nrc3265

[139] Christiansen JJ, Rajasekaran AK. Reassessing epithelial to mesenchymal transition as a prerequisite for carcinoma invasion and metastasis. Cancer Res. 2006;66:8319–26. 16951136 10.1158/0008-5472.CAN-06-0410

[140] Ye X, Brabletz T, Kang Y, Longmore GD, Nieto MA, Stanger BZ, et al. Upholding a role for EMT in breast cancer metastasis. Nature. 2017;547:E1-3. 28682326 10.1038/nature22816 PMC6283276

[141] Aiello NM, Brabletz T, Kang Y, Nieto MA, Weinberg RA, Stanger BZ. Upholding a role for EMT in pancreatic cancer metastasis. Nature. 2017;547:E7-8. 28682339 10.1038/nature22963 PMC5830071

[142] Guan X, Ma F, Li C, Wu S, Hu S, Huang J, et al. The prognostic and therapeutic implications of circulating tumor cell phenotype detection based on epithelial-mesenchymal transition markers in the first-line chemotherapy of HER2-negative metastatic breast cancer. Cancer Commun (Lond). 2019;39:1. 30606259 10.1186/s40880-018-0346-4 PMC6319003

[143] Schliekelman MJ, Taguchi A, Zhu J, Dai X, Rodriguez J, Celiktas M, et al. Molecular portraits of epithelial, mesenchymal and hybrid states in lung adenocarcinoma and their relevance to survival. Cancer Res. 2015;75:1789–800. 25744723 10.1158/0008-5472.CAN-14-2535 PMC4846295

[144] Hendrix MJ, Seftor EA, Seftor RE, Trevor KT. Experimental co-expression of vimentin and keratin intermediate filaments in human breast cancer cells results in phenotypic interconversion and increased invasive behavior. Am J Pathol. 1997;150:483–95. 9033265 PMC1858294

[145] Huang M-S, Fu L-H, Yan H-C, Cheng L-Y, Ru H-M, Mo S, et al. Proteomics and liquid biopsy characterization of human EMT-related metastasis in colorectal cancer. Front Oncol. 2022;12: 790096. 36249004 10.3389/fonc.2022.790096 PMC9560976

[146] Zhang Y, Men Y, Wang J, Xing P, Zhao J, Li J, et al. Epithelial circulating tumor cells with a heterogeneous phenotype are associated with metastasis in NSCLC. J Cancer Res Clin Oncol. 2022;148:1137–46. 34255149 10.1007/s00432-021-03681-9 PMC9016037

[147] Wei J, Deng W, Weng J, Li M, Lan G, Li X, et al. Epithelial-mesenchymal transition classification of circulating tumor cells predicts clinical outcomes in progressive nasopharyngeal carcinoma. Front Oncol. 2022;12: 988458. 36212389 10.3389/fonc.2022.988458 PMC9532596

[148] Qi L-N, Xiang B-D, Wu F-X, Ye J-Z, Zhong J-H, Wang Y-Y, et al. Circulating tumor cells undergoing EMT provide a metric for diagnosis and prognosis of patients with hepatocellular carcinoma. Can Res. 2018;78:4731–44. 10.1158/0008-5472.CAN-17-2459 29915159

[149] Yu M, Bardia A, Wittner BS, Stott SL, Smas ME, Ting DT, et al. Circulating breast tumor cells exhibit dynamic changes in epithelial and mesenchymal composition. Science. 2013;339:580–4. 23372014 10.1126/science.1228522 PMC3760262

[150] Li T-T, Liu H, Li F-P, Hu Y-F, Mou T-Y, Lin T, et al. Evaluation of epithelial-mesenchymal transitioned circulating tumor cells in patients with resectable gastric cancer: Relevance to therapy response. World J Gastroenterol. 2015;21:13259–67. 26715808 10.3748/wjg.v21.i47.13259 PMC4679757

[151] Zhao X-H, Wang Z-R, Chen C-L, Di L, Bi Z-F, Li Z-H, et al. Molecular detection of epithelial-mesenchymal transition markers in circulating tumor cells from pancreatic cancer patients: Potential role in clinical practice. World J Gastroenterol. 2019;25:138–50. 30643364 10.3748/wjg.v25.i1.138 PMC6328963

[152] Hu B, Tian X, Li Y, Liu Y, Yang T, Han Z, et al. Epithelial-mesenchymal transition may be involved in the immune evasion of circulating gastric tumor cells via downregulation of ULBP1. Cancer Med. 2020;9:2686–97. 32077634 10.1002/cam4.2871 PMC7163085

[153] Textor S, Fiegler N, Arnold A, Porgador A, Hofmann TG, Cerwenka A. Human NK cells are alerted to induction of p53 in cancer cells by upregulation of the NKG2D ligands ULBP1 and ULBP2. Cancer Res. 2011;71:5998–6009. 21764762 10.1158/0008-5472.CAN-10-3211

[154] Wei C, Yang C, Wang S, Shi D, Zhang C, Lin X, et al. Crosstalk between cancer cells and tumor associated macrophages is required for mesenchymal circulating tumor cell-mediated colorectal cancer metastasis. Mol Cancer. 2019;18:64. 30927925 10.1186/s12943-019-0976-4 PMC6441214

[155] Labelle M, Begum S, Hynes RO. Direct signaling between platelets and cancer cells induces an epithelial-mesenchymal-like transition and promotes metastasis. Cancer Cell. 2011;20:576–90. 22094253 10.1016/j.ccr.2011.09.009 PMC3487108

[156] Nieto MA. Epithelial plasticity: a common theme in embryonic and cancer cells. Science. 2013;342:1234850. 24202173 10.1126/science.1234850

[157] Luo Q, Wang C, Peng B, Pu X, Cai L, Liao H, et al. Circulating tumor-cell-associated white blood cell clusters in peripheral blood indicate poor prognosis in patients with hepatocellular carcinoma. Front Oncol. 2020;10:1758. 33224869 10.3389/fonc.2020.01758 PMC7667255

[158] Li Z, Fan L, Wu Y, Niu Y, Zhang X, Wang B, et al. Analysis of the prognostic role and biological characteristics of circulating tumor cell-associated white blood cell clusters in non-small cell lung cancer. J Thorac Dis. 2022;14:1544–55. 35693614 10.21037/jtd-22-423 PMC9186234

[159] Guan Y, Xu F, Tian J, Gao K, Wan Z, Wang Y, et al. The prognostic value of circulating tumour cells (CTCs) and CTC white blood cell clusters in patients with renal cell carcinoma. BMC Cancer. 2021;21:826. 34271857 10.1186/s12885-021-08463-7 PMC8285812

[160] Wang Z, Zhang P, Chong Y, Xue Y, Yang X, Li H, et al. Perioperative circulating tumor cells (CTCs), MCTCs, and CTC-white blood cells detected by a size-based platform predict prognosis in renal cell carcinoma. Dis Markers. 2021;2021:9956142. 34733376 10.1155/2021/9956142 PMC8560287

[161] Qiu Y, Zhang X, Deng X, Zhang R, Cai Z, Zhang Z, et al. Circulating tumor cell-associated white blood cell cluster is associated with poor survival of patients with gastric cancer following radical gastrectomy. Eur J Surg Oncol. 2022;48:1039–45. 34836729 10.1016/j.ejso.2021.11.115

[162] Guan X, Li C, Li Y, Wang J, Yi Z, Liu B, et al. Epithelial-mesenchymal-transition-like circulating tumor cell-associated white blood cell clusters as a prognostic biomarker in HR-Positive/HER2-negative metastatic breast cancer. Front Oncol. 2021;11: 602222. 34150608 10.3389/fonc.2021.602222 PMC8208036

[163] Szczerba BM, Castro-Giner F, Vetter M, Krol I, Gkountela S, Landin J, et al. Neutrophils escort circulating tumour cells to enable cell cycle progression. Nature. 2019;566:553–7. 30728496 10.1038/s41586-019-0915-y

[164] Peng D, Tanikawa T, Li W, Zhao L, Vatan L, Szeliga W, et al. Myeloid-derived suppressor cells endow stem-like qualities to breast cancer cells through IL6/STAT3 and NO/NOTCH cross-talk signaling. Cancer Res. 2016;76:3156–65. 27197152 10.1158/0008-5472.CAN-15-2528 PMC4891237

[165] Erpenbeck L, Schön MP. Deadly allies: the fatal interplay between platelets and metastasizing cancer cells. Blood. 2010;115:3427–36. 20194899 10.1182/blood-2009-10-247296 PMC2867258

[166] Palumbo JS, Talmage KE, Massari JV, La Jeunesse CM, Flick MJ, Kombrinck KW, et al. Platelets and fibrin(ogen) increase metastatic potential by impeding natural killer cell-mediated elimination of tumor cells. Blood. 2005;105:178–85. 15367435 10.1182/blood-2004-06-2272

[167] Nieswandt B, Hafner M, Echtenacher B, Männel DN. Lysis of tumor cells by natural killer cells in mice is impeded by platelets. Cancer Res. 1999;59:1295–300. 10096562

[168] Placke T, Örgel M, Schaller M, Jung G, Rammensee H-G, Kopp H-G, et al. Platelet-derived MHC class I confers a pseudonormal phenotype to cancer cells that subverts the antitumor reactivity of natural killer immune cells. Cancer Res. 2012;72:440–8. 22127925 10.1158/0008-5472.CAN-11-1872

[169] Ren J, He J, Zhang H, Xia Y, Hu Z, Loughran P, et al. Platelet TLR4-ERK5 axis facilitates NET-mediated capturing of circulating tumor cells and distant metastasis after surgical stress. Cancer Res. 2021;81:2373–85. 33687949 10.1158/0008-5472.CAN-20-3222 PMC8137664

[170] Najmeh S, Cools-Lartigue J, Rayes RF, Gowing S, Vourtzoumis P, Bourdeau F, et al. Neutrophil extracellular traps sequester circulating tumor cells via *β*1-integrin mediated interactions. Int J Cancer. 2017;140:2321–30. 28177522 10.1002/ijc.30635

[171] Liu X, Song J, Zhang H, Liu X, Zuo F, Zhao Y, et al. Immune checkpoint HLA-E:CD94-NKG2A mediates evasion of circulating tumor cells from NK cell surveillance. Cancer Cell. 2023;41:272-287.e9. 36706761 10.1016/j.ccell.2023.01.001

[172] Tanaka A, Sakaguchi S. Regulatory T cells in cancer immunotherapy. Cell Res. 2017;27:109–18. 27995907 10.1038/cr.2016.151 PMC5223231

[173] Lu Y, Lian S, Ye Y, Yu T, Liang H, Cheng Y, et al. S-Nitrosocaptopril prevents cancer metastasis in vivo by creating the hostile bloodstream microenvironment against circulating tumor cells. Pharmacol Res. 2019;139:535–49. 30366102 10.1016/j.phrs.2018.10.020

[174] Deutsch TM, Riethdorf S, Fremd C, Feisst M, Nees J, Fischer C, et al. HER2-targeted therapy influences CTC status in metastatic breast cancer. Breast Cancer Res Treat. 2020;182:127–36. 32436146 10.1007/s10549-020-05687-2 PMC7274999

[175] Mu W, Chu Q, Yang H, Guan L, Fu S, Gao T, et al. Multipoint costriking nanodevice eliminates primary tumor cells and associated-circulating tumor cells for enhancing metastasis inhibition and therapeutic effect on HCC. Adv Sci (Weinh). 2022;9:2101472. 35356152 10.1002/advs.202101472 PMC8948568

[176] Yao J, Feng J, Gao X, Wei D, Kang T, Zhu Q, et al. Neovasculature and circulating tumor cells dual-targeting nanoparticles for the treatment of the highly-invasive breast cancer. Biomaterials. 2017;113:1–17. 27794222 10.1016/j.biomaterials.2016.10.033

[177] Allard WJ, Matera J, Miller MC, Repollet M, Connelly MC, Rao C, et al. Tumor cells circulate in the peripheral blood of all major carcinomas but not in healthy subjects or patients with nonmalignant diseases. Clin Cancer Res. 2004;10:6897–904. 15501967 10.1158/1078-0432.CCR-04-0378

